# Prevalence of Enuresis in Children, Adolescents, and Young Adults Diagnosed With Attention Deficit Hyperactivity Disorder

**DOI:** 10.7759/cureus.55073

**Published:** 2024-02-27

**Authors:** Basmah Aljabri, Joud AlSolami, Ameerah Alnakhli, Arwa Ahmed, Abrar Alsharief

**Affiliations:** 1 Pediatrics Department, King Abdulaziz University, Faculty of Medicine, Jeddah, SAU; 2 Autism Centre, King Faisal Specialist Hospital and Research Centre, Riyadh, SAU; 3 Pediatircs Department, King Abdulaziz University, Faculty of Medicine, Jeddah, SAU

**Keywords:** voiding dysfunction, pediatrics, neurodevelopmental disorders, enuresis, adhd

## Abstract

Background

Enuresis, or bedwetting, is a common condition affecting millions of children worldwide. This can be a source of distress for both children and their families. Children, adolescents, and young adults with attention deficit hyperactivity disorder (ADHD) are at risk of developing enuresis. They have difficulties with executive functioning, including impulse control and emotional regulation. These difficulties may contribute to the development of enuresis, as individuals may struggle to recognize the urge to use the bathroom or have difficulty controlling their bladder during sleep.

Objective

To assess the prevalence of enuresis in children, adolescents, and young adults with ADHD and determine whether the presence of other behavioral disorders such anxiety, depression, learning disabilities, and autism comorbid with ADHD is a risk factor for developing enuresis.

Method

A case-control study included 213 children, adolescents, and young adults aged seven to 23 years, with 139 males and 74 females. A total of 161 participants were diagnosed with ADHD. Data collection consisted of a semi-structured interview conducted with each participant or their parents in person during their visit to Developmental Pediatric Clinics and Psychiatry Clinics. The questions were designed to collect data on the participant's ADHD diagnosis, enuresis history, other behavioral disorders, such as anxiety, depression, and learning difficulties, and any relevant medical or developmental history. The interview lasted approximately 30 minutes.

Results

Children, adolescents, and young adults with ADHD had a significantly higher prevalence of enuresis than the control group (13.6% vs. 0.9%, p = 0.01). Other behavioral disorders comorbid with ADHD did not pose a statistically significant risk for the development of enuresis (p = 0.36).

Conclusions

This study supports that children, adolescents, and young adults diagnosed with ADHD are more likely to have enuresis than those without ADHD. This finding is consistent with previous research and emphasizes the importance of a thorough evaluation and comprehensive treatment plan for individuals with ADHD.

## Introduction

Bedwetting, also known as enuresis, is involuntary urination during sleep. It is a common problem in children but can also occur in adults. Most children outgrow bedwetting by the time they are seven years old [1]. Locally, the prevalence of nocturnal enuresis among normal children in the eastern region of Saudi Arabia aged 3-15 years was estimated to be 31.2% [2]. Recent research has suggested a link between enuresis and Attention Deficit Hyperactivity Disorder (ADHD), which is defined as a neurodevelopmental disorder that affects how a person pays attention, controls impulsive behaviors, and hyperactivity [3]. It has been found that children with enuresis have a higher prevalence of ADHD and externalizing problems [4]. A cross-sectional study conducted in Makkah, Saudi Arabia, in 2022-2023, found that the prevalence of ADHD diagnosis in children aged 4-14 years old is 52.5% [5]. Another systematic review showed that the epidemiology of ADHD in Arab countries ranged between 1.3-16% [6]. Different reasons can be accounted for the disparate prevalence of ADHD reported from different countries in the same region. First, the definition periods for the prevalence, i.e., point prevalence vs. 12-month prevalence.

Second, the differences in the age range included in the studies. Third, the diagnostic criteria used for ADHD diagnosis, as this systematic review included studies from 1978 to 2014, where the Diagnostic Statistical Manual of Mental Disorders (DSM) had different revisions and updates. Finally, although they were from the same region, the population's ethnicity, and biological factors differed. 

In a case-control study done in 2020, the prevalence of ADHD in children diagnosed with enuresis was estimated to be 40% [7]. Moreover, the combination of enuresis and ADHD increases the possibility of other comorbid psychological disorders than patients diagnosed only with ADHD [7]. Children with ADHD often have difficulty with executive functioning, including impulse control and emotional regulation. These difficulties may contribute to the development of enuresis, as they struggle to recognize the urge to use the bathroom or have difficulty controlling their bladder during sleep [8]. While previous studies have investigated the association between ADHD and daytime urinary incontinence, the proposed case-control study will measure the prevalence of enuresis in ADHD patients in the western region of Saudi Arabia and assess whether the presence of other behavioral disorders comorbid with ADHD is a risk factor for developing enuresis.

## Materials and methods

This case-control study was conducted at King Abdulaziz University Hospital (KAUH) in Jeddah, Saudi Arabia, from July 2022 to July 2023. The Unit of Biomedical Ethics, Research Ethics Committee (REC) approved conducting the project, with reference number 394-22.

The inclusion criteria were children, adolescents, and young adults with a confirmed medical diagnosis of ADHD. ADHD was diagnosed either by a developmental pediatrician or by a child and adolescent psychiatrist, using the DSM5 criteria, which mandate the presence of the disease symptoms that include inattention and or hyperactivity-impulsivity, or both for more than six months continuously, and the symptoms must be present in two different environments (at home and school) [3]. These symptoms are not caused by organic problems and must be present before the age of 12 years and older than age four years. The Vanderbilt ADHD Diagnostic Parent and Teacher Rating Scales (VADRS) were used to measure the symptoms of ADHD and their effects on children's behaviors and academic performance.

Exclusion criteria were those with (1) congenital anomalies of the urinary system, (2) previous pelvic surgery, (3) recent history of urinary tract infection, (4) individuals with intellectual disability, (5) organic central nervous system diseases, and (6) the current use of medications known to interfere with bladder or sphincter function, such as anticholinergics, diuretics, antidepressants, and antipsychotics.

Participants were 213 children, adolescents, and young adults aged seven to 23 years, with 139 males and 74 females. A total of 161 participants were diagnosed with ADHD. 

Data collection

Data collection consisted of a semi-structured interview conducted in-person with each participant or participant's parents by data collectors, during their routine visits to Developmental Pediatric Clinics and Psychiatry Clinics. The questions were related to information on: the participant's ADHD diagnosis, enuresis history, and other co-morbid conditions associated with ADHD diagnosis such as anxiety, depression, and learning disability (see Appendices). Also, any relevant medical or developmental history. The interview lasted approximately 30 minutes.

Statistical analysis

GraphPad Prism 8.0.2 (GraphPad Software Inc., San Diego, USA) was used to analyze the collected data statistically, calculating descriptive statistics and p-values using the chi-square test and t-test. A p-value less than 0.05 was considered as significant.

## Results

Two hundred thirteen patients were included in the study, with 139 (65%) males and 74 (35%) females aged seven to 23 years. The number of participants in the control group was 211. The control group was recruited from the community, schools, and universities. Participants diagnosed with ADHD were 161 (75.5%), 35 (21.7%) of those with ADHD were diagnosed with enuresis, 124 (77%) of ADHD patients were diagnosed with co-existing conditions. The presence of enuresis in children, adolescents, and young adults with ADHD was higher than in the control group (21.7 % vs. 0.9%) (Table 1).

**Table 1:** Prevalence of co-existing conditions and enuresis in the participants Data presented as: N: Number, %: percentage ADHD: attention deficit hyperactivity disorder

Variable	ADHD Diagnosed (n = 161) N (%)	Control group (n = 211) N (%)	P value
Co-existing conditions	124 (77%)	15 (7.1%)	<0.0001
Enuresis	35 (21.7%)	2 (0.9%)	0.0095

The average age of all participants (mean; SD) was 14.79±7.815. Age when the patients diagnosed with ADHD was 6.726±2.420; when diagnosed with enuresis, it was 7.093±4.492; and when diagnosed with co-existing conditions, it was 7.814±6.652 (Table 2).

**Table 2:** Average age of patients at different disease diagnoses Data presented as Mean, SD: Standard Deviation ADHD: attention deficit hyperactivity disorder

Age (yrs)	mean±SD
General Age	14.79±7.815
Age at ADHD diagnosis	6.726±2.420
Age at Enuresis Diagnosis	7.093±4.492
Age at Coexisting Condition Diagnosis	7.814±6.652

The total group with ADHD was 161 participants, 124 (77%) of them had co-morbid conditions, while those with ADHD and enuresis were 35 (21.7%), and 24 (68.5%) of them had co-morbid conditions. The co-morbid conditions are higher among participants with ADHD and enuresis than with ADHD only, as shown in Table 3.

**Table 3:** Comparison of co-existing conditions in the participants with ADHD alone and in ADHD with enuresis Data presented as: N: Number, %: percentage ADHD: attention deficit hyperactivity disorder

Variable	ADHD group: N (%)	ADHD and Enuresis group: N (%)	P value
				Co-existing conditions	124 (77%)	24 (68.5%)	0.3668

Among all the co-morbid conditions, learning disabilities are the most common condition in children, adolescents, and young adults with ADHD, affecting over one-third - 75 (35.2%) individuals in this group. It is followed by autism spectrum disorder being the second most common co-existing condition, affecting over one-fifth: 49 (23.0%) of individuals with ADHD. Anxiety disorder and depression are also common co-comorbid conditions, affecting over 33 (15%) and 18 (8%) of individuals with ADHD, respectively (Table 4).

**Table 4:** The most common co-existing conditions in children and adolescents with ADHD and enuresis Data presented as frequency (number) and percentage (%)

Co-existing conditions	Frequency (N)	Percentage (%)
Learning disabilities	75	35.2
Autism Spectrum Disorder	49	23
Anxiety disorder	33	15.4
Depression	18	8.4

## Discussion

Generally, this study's overall prevalence of enuresis was estimated to be 16.3% in both genders, with a higher prevalence in males (11.7%) than in females (4.6%). Compared to other studies done locally, Alhifthy et al. [2] reported a higher prevalence of enuresis by 48.3% in the Eastern province of Saudi Arabia, in a younger age group from 3 to 12 years. Meanwhile, in Jazan province, Sherah et al. reported a substantially greater prevalence of enuresis by 52.6%, in the younger age group from 5-12 years [9]. An overall estimate of enuresis in Saudi Arabia was 76.4% in the age group from 6 to 12 years [10]. A lower prevalence in our study compared to the other local studies can be attributed to the smaller sample size and the different age range. Another explanation for a lower prevalence in this study is the setup. This study is a hospital-based cohort, while the others were population-based cohorts. 

Previous research indicated that individuals with ADHD have a higher risk of developing enuresis and other behavioral and developmental disorders [5,7]. Similarly, the results of this study support the same international findings that children, adolescents, and young adults with ADHD have a significantly higher prevalence of enuresis and co-existing conditions than the control group. The Yang study [11] described broader lower urinary tract symptoms, not only enuresis, such as urgency, intermittency, and urinary incontinence, being also more prevalent in school-aged individuals with ADHD. It is essential for all health professionals who are involved in the care of individuals with ADHD to screen for the presence of enuresis and other lower urinary tract symptoms, along with the most common co-morbid conditions.

The association between ADHD and enuresis is of unknown etiology [1]. Different hypotheses related to the brain stem and cortex functions have been postulated. An arousal deficit secondary to delayed functional maturation of the brain stem in enuretic children has been strongly supported [12]. Another hypothesis related to uncoordinated central adrenergic stimulation with ADHD causing sphincter overactivity, with subsequent enuresis [13]. Lastly, the behavioral challenges associated with ADHD, including impulsivity, hyperactivity, and lack of attention, lead to an inappropriate response to the desire for micturition [14]. Despite the high heritability in the presence of ADHD and enuresis, the limited genetic studies do not support genetic factors as underlying etiology in the association between ADHD and enuresis [15]. 

Nowadays, it is well known that there is a strong association between ADHD and co-morbid conditions [16]. Sometimes, the co-existing conditions come to the forefront of the disorder as a vital part of the complex neurodevelopmental disorder [17]. In our sample, the group with ADHD and enuresis has a higher incidence of co-morbid conditions than the group with ADHD alone. Learning disability was the most common co-morbid condition, 35.2%; this is close to the international figures, where about 10%-95% of ADHD children meet the criteria for a learning disorder [18]. This discrepancy may relate to differences in the diagnostic assessment of learning disability. Internalizing disorders such as depression and anxiety are also commonly present with ADHD (12%-50%) and (15%-35%), respectively [16]. In this study, 8.4% were affected with depression and 15.4% with anxiety disorder. These smaller percentages are related to a smaller sample size.

This study has some limitations. First, it was conducted at a single site, limiting the findings' generalizability. Second, the sample size was relatively small, which may have limited the ability to detect statistically significant differences between the groups. Third, the study was case-control and thus cannot establish causality, and further research is needed to explore it. Fourth, since it was an interview-based study, there could be a possibility of recall bias.

## Conclusions

This study demonstrates that enuresis and other behavioral disorders are more common in children, adolescents, and young adults with ADHD in this area than in those without ADHD. The findings have several implications for clinical practice. First, clinicians should be aware of the high prevalence of enuresis and co-existing behavioral conditions in individuals with ADHD. Second, clinicians should screen individuals of all age groups with ADHD for enuresis and co-existing behavioral conditions so that early diagnosis and treatment can be initiated. Third, clinicians should develop individualized treatment plans for children, adolescents, and young adults with ADHD that are associated with co-existing conditions or enuresis, taking into account the unique needs of each person. Fourth, proper management of ADHD symptoms and co-morbid conditions can help in the management of enuresis. 

## Appendices

**Table 5:** Questionnaire used in the study ADHD: attention deficit hyperactivity disorder

Does the patient agree to participate in the Research?	Yes No	
Patient number		
Patient name		
Date of birth		
Contact phone number		
Is the patient diagnosed with ADHD?	Yes No	
How old was the patient when first diagnosed with ADHD?		
Coexisting conditions:	Autism Learning disabilities Depression Anxiety Tic disorder Oppositional disorder Conduct disorder Others: specify	
How old was the patient when diagnosed with each coexisting condition?		
Is the patient diagnosed with Enuresis?	Yes No The patient is not diagnosed but the symptoms occur twice a week for more or equal to 3 months	
How old was the patient when diagnosed with Enuresis?		
Type of Enuresis:	Primary Secondary Diurnal Nocturnal	
